# Low muscle mass and Charlson comorbidity index are risk factors for short-term postoperative prognosis of elderly patients with gastrointestinal tumor: a cross-sectional study

**DOI:** 10.1186/s12877-021-02683-z

**Published:** 2021-12-23

**Authors:** Jiaqiu Wang, Liqian Xu, Shunmei Huang, Quan Hui, Xuexue Shi, Qin Zhang

**Affiliations:** 1 Department of Geriatrics, The First Affiliated Hospital, Zhejiang University School of Medicine, 79 Qingchun Raod, Hangzhou Zhejiang, 310003 People’s Republic of China; 2 Zhejiang Provincial Key Laboratory for Diagnosis and Treatment of Aging and Physic-chemical Injury Diseases, The First Affiliated Hospital, Zhejiang University School of Medicine, 79 Qingchun Raod, Hangzhou Zhejiang, 310003 People’s Republic of China

**Keywords:** Low muscle mass, Gastrointestinal tumor, Geriatrics, Complications, Length of stay

## Abstract

**Background:**

Sarcopenia is one of the most frequent syndromes in older adults and one of its main characteristics is low muscle mass. Gastrointestinal tumor is a malignant disease with high incidence. This study aimed to investigate the risk factors of low muscle mass in older adults with gastrointestinal tumor, the prognostic indicators of and short-term outcomes after resection for gastrointestinal tumor, and to explore the relationship between low muscle mass and short-term postoperative prognosis.

**Method:**

A total of 247 older patients with gastrointestinal tumors who underwent radical resection in 2019 were included in this study. Relevant indexes were calculated using L3 slice image of computed tomography (CT) to evaluate low muscle mass. Short-term postoperative complications and length of stay were considered as short-term outcomes of this study.

**Results:**

Advanced age, lower higher body mass index (BMI), lower hemoglobin, having history of abdominal surgery and higher visceral fat index (VFI) were risk factors of low muscle mass, while higher BMI and lower subcutaneous fat index (SFI) were protective factors of low muscle mass. Further multivariate logistic regression analysis showed that having history of abdominal surgery, advanced age and lower BMI were independent risk factors. Low muscle mass and higher Charlson comorbidity index were independent risk factors of short-term postoperative complications in older adults with gastrointestinal tumor. Higher Charlson comorbidity index gave rise to longer length of stay.

**Conclusions:**

Low muscle mass and higher Charlson comorbidity index predict poor short-term prognosis of older patients undergoing gastrointestinal tumor resection.

## Introduction

With the aging tendency of global population, the number of people with low muscle mass is increasing. Low muscle mass is one of the main characteristics of sarcopenia which is one of the most frequent syndromes in older adults. As newly released guideline, sarcopenia is defined as the decline in muscle quality, strength and function which are relevant with aging [1]. Older adults with sarcopenia have a higher risk of falls, fractures, and motor functional decline. In recent years, more and more studies on low muscle mass have been conducted to help explore sarcopenia, and guidelines about sarcopenia have been constantly updated at home and abroad to help clinical work and related scientific research. As previous research showed, nearly 1/10 older adults suffered with muscle quality decline [2], so that the society should attach more importance to sarcopenia.

Solid tumor of gastrointestinal tract is one of the malignant diseases of high incidence. According to the GLOBOCAN 2018 data released by the World Health Organization (WHO) in 2018, colorectal cancer ranks the third and gastric cancer the fourth among the top ten tumors in terms of incidence [3]. There are about 1.4 million new cases of colorectal cancer and 1 million new cases of stomach cancer per year globally, and about 70% of stomach cancer occurs in developing countries [4]. In China, there are a large number of patients with colorectal tumor and stomach tumor, and the effective treatment for most of them potentially is surgical resection. Older adults with cancer have a higher incidence of sarcopenia [4]. When both cancer and sarcopenia happen to older patients, it may increase the clinical adverse events, accelerate malignancy process and further influence the survival of older patients [4, 5]. Recent studies have showed that low muscle mass is correlated with poor prognosis after resection surgery for colorectal cancer and stomach cancer [6, 7]. However, relevant research in the Chinese population is still limited.

Therefore, this study explored the relationship between low muscle mass, common clinical indicators and short-term postoperative complications in older patients with colorectal cancer and gastric cancer. We aimed to further identify prognostic indicators for older patients with resection and to intervene precisely.

## Method

### Patients

Older patients undergoing resection for gastric cancer or colorectal cancer at the First Affiliated Hospital of Zhejiang University from January to December in 2019 were included in this cross-sectional study. Inclusion criteria were: 1. patients’ age ≥ 70 years old; 2. Abdominal CT were completed within 15 days before the surgery; 3. Postoperative pathology confirmed malignant tumor. Patients with incomplete important data, such as leukocyte, hemoglobin, neutrophils to lymphocytes ratio (NLR), albumin, height, weight, postoperative course record and etc., were excluded from this study.

All the data were collected by experienced geriatrician from electronic medical records, which included: basic information such as sex, height, weight, age and etc.; comorbidities were evaluated by Charlson comorbidity index, the gastrointestinal tumor treated in this hospital stay was not evaluated as comorbidity [8]; history of abdominal surgery related to malignancy or benign diseases; history of alcohol, tobacco; laboratory parameters included leukocyte, hemoglobin, albumin and NLR within 15 days before the surgery; CT slice at L3; the operation data included the duration of operation, intraoperative blood loss, and postoperative pathology; cancer stage which was based on The Union for International Cancer Control (UICC) cancer tumor node metastasis (TNM) staging system (8th edition) [9]; and postoperative outcomes included postoperative complications which was evaluated by Clavien-Dindo classification [10] and length of stay.

### Imaging analysis

**Fig. 1:**
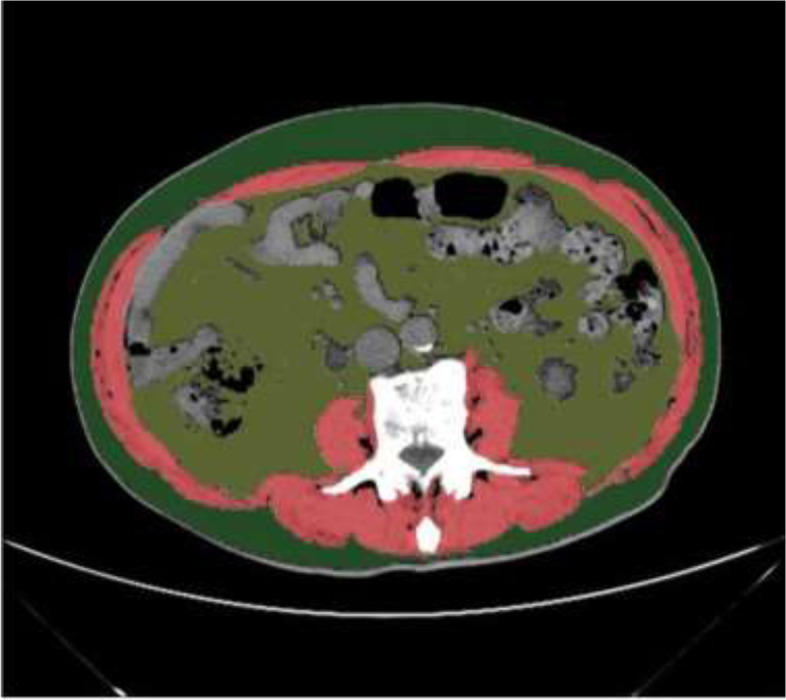
Skeletal muscle and fat tissue in L3

The CT slice at L3 was analysed by Image J (NIH Image J version 1.52a). Referred to the former research, skeletal muscle threshold is -29HU to 150HU and the adipose tissue threshold is -190HU to -30HU [6]. An example is shown in Fig. 1, in which the red part represents muscle tissue, the dark green part represents subcutaneous fat tissue, and the light green part represents visceral fat tissue. This study measured the skeletal muscle area (SMA), subcutaneous fat areas (SFA) and visceral fat area (VFA). Obtained area values were divided by the square of the patient’s height (m^2^) to get skeletal muscle index (SMI), SFI and VFI. Visceral-to-subcutaneous ratio of fat area (VSR) was also calculated as a parameter. According to the previous large sample Chinese population study, male SMI ≤ 40.8 cm^2^/m^2^ and female SMI ≤ 34.9 cm^2^/m^2^ were defined as low muscle mass [11].

### Statistics

Data are given as means (with standard deviation) or medians (with interquartile range). Univariate and multivariate logistic regression were performed to analyze the related variables of postoperative complications. Results were showed as odds ratios (ORs) with 95% confidence intervals (CIs). Linear regression was performed to analyze the related factors for length of stay. IBM SPSS 25 was used for all this analysis.

## Results

### Patients characteristics

The characteristics of included patients were presented in Table 1. A total of 247 older patients undergoing gastrointestinal tumor surgery were included in this study, 163 of them were men. Ninety-eight patients were suffering from gastric cancer and the rest from colorectal cancer. Their average age was about 75.5 and their average BMI was around 22.2 kg/m^2^. Fifty-two patients had history of alcohol and 68 patients had history of tobacco and most of them were male. Sixty patients had history of abdominal surgery. The mean values of leukocyte, hemoglobin, albumin and NLR were within the range of normal clinical values. Most of the patients were in TNM stage I and II, but only 13 patients were defined as well differentiated histologic type.

**Table 1:** Overall characteristics of patients

	gastric cancer (*n* = 99)	colorectal cancer (*n* = 148)	Total(*n* = 247)
Age (years)	74.6(4.1)	76.1(5.1)	75.5(4.8)
Male, n(%)	77(77.8)	86(58.1)	163(65.6)
BMI (kg/m^2^)	21.8(2.9)	22.5(3.4)	22.2(3.2)
Charlson comorbidity index	4(1)	4(2)	4(2)
History of alcohol, n(%)	28(28.2)	24(16.2)	52(21.1)
History of tobacco, n(%)	40(40.4)	28(18.9)	68(27.5)
History of abdominal surgery, n(%)	15(15.1)	45(30.4)	60(24.3)
Major laboratory indicators			
Leukocyte(×10^9/L)	5.4(2.0)	5.9(2.2)	5.7(2.1)
Hemoglobin(g/L)	125.0(28.8)	129.0(33.1)	126.5(32.0)
Albumin(g/L)	39.7(8.2)	43.0(5.4)	42.1(6.7)
NLR	2.3(1.8)	2.4(1.4)	2.4(1.5)
Intraoperative blood loss (mL)	132.8(81.9)	107.6(93.1)	116.3(89.0)
Duration of operation (min)	202.0(67.0)	180.0(63.0)	188(65)
Cancer stage, n(%)			
I	56(56.6)	42(28.4)	98(39.7)
II	32(32.3)	51(34.5)	83(33.6)
III	11(11.1)	55(37.2)	66(26.7)
Histologic type, n(%)			
Poorly differentiated	63(63.6)	36(24.3)	99(40.1)
Moderately differentiated	29(29.3)	106(71.6)	135(54.7)
Well differentiated	7(7.1)	6(4.1)	13(5.3)
Body composition			
SMI (cm^2^/m^2^)	44.7(9.4)	40.9(10.7)	42.6(10.9)
SFI (cm^2^/m^2^)	34.5(19.2)	39.7(27.6)	37.6(24.3)
VFI (cm^2^/m^2^)	45.7(28.6)	50.7(41.0)	49.4(35.7)
VSR	1.3(0.7)	1.2(0.8)	1.5(0.8)
Low muscle mass, n(%)	22(22.2)	49(33.1)	71(28.7)
Postoperative complication, n(%)	14(14.1)	43(29.1)	62(25.1)
Length of stay (day)	10(3)	8(4)	9(5)

*BMI* body mass index, *NLR* neutrophil to lymphocyte ratio, *SMI* skeletal muscle index, *SFI* subcutaneous fat index, *VFI* visceral fat index, *VSR* visceral to subcutaneous ratio of fat area

Using the ImageJ software, we calculated the parameters of human body composition. SMI, SFI, VFI and VSR were 42.6 ± 10.9 cm^2^/m^2^, 37.6 ± 24.3 cm^2^/m^2^, 49.4 ± 35.7 cm^2^/m^2^, 1.5 ± 0.8.

### Factors associated with low muscle mass

Seventy-one patients were classified as low muscle mass. The incidence rate of low muscle mass in this study was about 28.7%. Twenty-two of them (31.0%) were with gastric tumor and 49 (69.0%) were with colorectal tumor.

This study detected several factors associated with low muscle mass as Table 2 showed. In univariate analysis, low muscle mass was associated with advanced age (*p*<0.001), lower BMI (*p*<0.001), lower hemoglobin (*p* = 0.009), having history of abdominal surgery (*p* = 0.010), and higher VFI (*p*<0.001). Higher BMI (*p*<0.001) and lower SFI (*p* = 0.005) were protective factors for low muscle mass. Multivariate logistic regression was used to further analyze the data and it showed that having history of abdominal surgery (OR:2.5; 95% CI:1.3 to 5.3; *p* = 0.007), advanced age (OR:3.0; 95% CI:1.6 to 5.7; *p* = 0.001) and lower BMI (OR:3.4; 95% CI:1.3 to 8.6; *p* = 0.011) were independent risk factors for low muscle mass.

**Table 2:** Univariate and multivariate logistic regression analysis of low muscle mass associated factors

Variable	Univariate analysis			Multivariate analysis		
	OR	95%CI	*p* Value	OR	95%CI	*p* Value
Sex^a^	0.6	0.3–1.1	0.084			
Age^b^	3.2	1.8–5.8	<0.001	3.0	1.6–5.7	0.001
Cancer site^c^	0.6	0.3–1.1	0.078			
BMI^d^	3.3	1.3–8.0	0.009	3.4	1.3–8.6	0.011
BMI^d*^	0.2	0.1–0.4	<0.001	0.2	0.1–0.4	<0.001
Charlson comorbidity index^e^	1.5	0.8–2.7	0.207			
History of alcohol^f^	1.7	0.8–3.1	0.086			
History of tobacco^g^	1.5	0.8–2.8	0.252			
History of abdominal surgery^h^	2.2	1.2–4.1	0.010	2.6	1.3–5.3	0.007
Leukocyte^i^	1.4	0.8–2.4	0.254			
Hemoglobin^j^	2.1	1.2–3.7	0.009			
Albumin^k^	1.7	0.9–2.9	0.075			
NLR^l^	0.7	0.4–1.2	0.159			
Cancer stage^m^	1.1	0.8–1.5	0.470			
Histologic type^n^	1	0.6–1.7	0.860			
SFI^o^	0.4	0.3–0.8	0.005			
VFI^p^	2.9	1.6–5.3	<0.001			
VSR^q^	1.0	0.6–1.7	0.985			

*OR* odds ratio, *CI* confidence interval, *BMI* body mass index, *NLR* neutrophil to lymphocyte ratio, *SMI* skeletal muscle index, *SFI* subcutaneous fat index, *VFI* visceral fat index, *VSR* visceral to subcutaneous ratio of fat area

^a^female as reference

^b^age ≤ 75 as reference

^c^colorectal cancer as reference

^d^BMI<18.5 kg/m^2^ vs 18.5 kg/m^2^ ≤ BMI ≤ 24 kg/m^2^

^d*^BMI>24 kg/m^2^ vs 18.5 kg/m^2^ ≤ BMI ≤ 24 kg/m^2^

^e^Charlson comorbidity index≤4 as reference

^f^no history of alcohol as reference

^g^no history of tobacco as reference

^h^no history of abdominal surgery as reference

^i^leukocyte ≥ 5.7 × 10^9/L as reference

^j^hemoglobin ≥ 126.5 g/L as reference

^k^albumin ≥ 42.1 g/L as reference

^l^NLR ≥ 2.4 as reference

^m^stageII and stageIII vs stageI

^n^moderately and well differentiated vs poorly differentiated

^o^SFI ≤ 37.6 cm^2^/m^2^ as reference

^p^VFI ≤ 49.4 cm^2^/m^2^ as reference

^q^VSR ≤ 1.45 as reference

### Relationship between low muscle mass and postoperative complication

About 62 patients had grade two and above of Clavien-Dindo classification of surgical complications. The main complications included postoperative hemorrhage, pulmonary infection, abdominal infection, seroperitoneum, delirium, anastomotic leakage, ileus, venous thrombosis, heart failure, etc. Exploratory laparotomy was performed in four patients after operation because of the postoperative hemorrhage or ileus. One patient got interventional operation for multiple thrombosis. One patient died because of hemorrhoea. One patient discharged from hospital giving up treatment because of severe infection and bone marrow suppression.

Several factors were found associated with postoperative complications as Table 3 presented. In univariate analysis, advanced age (*p* = 0.012), higher Charlson comorbidity index (*p* = 0.014), and low muscle mass (*p* = 0.001) were associated with postoperative complications. Further multivariate logistic regression analysis showed that low muscle mass (OR:2.6; 95% CI:1.4 to 4.9; *p* = 0.002), and higher Charlson comorbidity index (OR:2.1; 95% CI:1.1 to 3.9; *p* = 0.026) were independent risk factors of postoperative complications.

**Table 3:** Univariate and multivariate logistic regression analysis of postoperative complications associated factors

Variable	Univariate analysis			Multivariate analysis		
	OR	95%CI	*p* Value	OR	95%CI	*p* Value
Sex^a^	1.4	0.8–2.6	0.226			
Age^b^	2.1	1.2–3.8	0.012			
Cancer site^c^	1.7	0.9–3.1	0.095			
BMI^d^	2.2	0.9–5.4	0.097			
BMI^d*^	0.8	0.4–1.6	0.618			
Charlson comorbidity index^e^	2.2	1.2–4.0	0.014	2.1	1.1–3.9	0.026
History of alcohol^f^	2.2	0.9–4.9	0.067			
History of tobacco^g^	1.1	0.6–2.1	0.723			
History of abdominal surgery^h^	1.8	1.0–3.5	0.065			
Leukocyte^i^	1.3	0.7–2.3	0.400			
Hemoglobin^j^	1.2	0.7–2.1	0.207			
Albumin^k^	0.9	0.5–1.7	0.851			
NLR^l^	0.7	0.4–1.2	0.207			
Cancer stage^m^	1.8	1.0–3.3	0.060			
Histologic type^n^	1.2	0.7–1.9	0.510			
Low muscle mass ^o^	2.7	1.5–5.0	0.001	2.6	1.4–4.9	0.002
SFI^p^	0.8	0.5–1.4	0.442			
VFI^q^	0.9	0.5–1.6	0.696			
VSR^r^	1.4	0.8–2.4	0.288			
Intraoperative blood loss^s^	1.4	0.7–2.6	0.386			
Duration of operation^t^	1.8	1.0–3.2	0.051			

*OR* odds ratio, *CI* confidence interval, *BMI* body mass index, *NLR* neutrophil to lymphocyte ratio, *SMI* skeletal muscle index, *SFI* subcutaneous fat index, *VFI* visceral fat index, *VSR* visceral to subcutaneous ratio of fat area

^a^female as reference

^b^age ≤ 75 as reference

^c^colorectal cancer as reference

^d^BMI<18.5 kg/m^2^ vs 18.5 kg/m^2^ ≤ BMI ≤ 24 kg/m^2^

^d*^BMI>24 kg/m^2^ vs 18.5 kg/m^2^ ≤ BMI ≤ 24 kg/m^2^

^e^Charlson comorbidity index≤4 as reference

^f^no history of alcohol as reference

^g^no history of tobacco as reference

^h^no history of abdominal surgery as reference

^i^leukocyte ≥ 5.7 × 10^9/L as reference

^j^hemoglobin ≥ 126.5 g/L as reference

^k^albumin ≥ 42.1 g/L as reference

^l^NLR ≥ 2.4 as reference

^m^stageII and stageIII vs stageI

^n^moderately and well differentiated vs poorly differentiated

^o^without low muscle mass as reference

^p^SFI ≤ 37.6 cm^2^/m^2^ as reference

^q^VFI ≤ 49.4 cm^2^/m^2^ as reference

^r^VSR ≤ 1.45 as reference

^s^blood loss≤116 mL as reference

^t^time ≤ 196 min as reference

### Factors associated with length of stay

Linear regression was employed to analyze the risk factors of length of stay. After the factors which were correlated with each other were excluded, it was found that Charlson comorbidity index (*p* = 0.019), tumor site (*p* = 0.016), and duration of surgery (*p* = 0.045) were significantly correlated with length of hospital stay. Higher Charlson comorbidity index and longer operative time will result in longer hospital stays. The length of stay of patient with gastric cancer was significantly longer than those who with colorectal cancer.

## Discussion

This study mainly investigated the risk factors of low muscle mass in older patients with digestive tract tumors and the relationship between low muscle mass and short-term postoperative outcome. This study found that the incidence of low muscle mass in older patients with gastric cancer or colorectal cancer was about 28.7%, which was clearly associated with history of abdominal surgery, lower BMI, and advanced age. The occurrence of postoperative complications was correlated with low muscle mass and higher Charlson comorbidity index.

The Asian Working Group for Sarcopenia (AWGS) released the latest expert consensus on the diagnosis and treatment of sarcopenia in 2019. As the expert consensus suggested, the commonly used methods for muscle mass measurement are DXA or BIA, and CT is also recognized as a good method for skeletal muscle mass measurement especially for assessing muscle volume [1, 12]. However, there is some debates on the diagnostic threshold. In general studies, L3 plane was selected to calculate the muscle area and SMI value. Low muscle mass was defined by the cut-off value of SMI. A study published in The Lancet Oncology in 2008 suggested that men with SMI < 52.4 cm^2^/m^2^ and women with SMI < 38.5 cm^2^/m^2^ were considered to have CT-assessed sarcopenia [13]. This cut-off value is frequently used. Different ethnic groups suit different SMI cut-off value and some studies chose more complicated cut-off values according to both BMI value and SMI value [14]. Since the object of this study was Asian population, and there is a big difference in physique between the western population and Asian population, we referred to a dependable study of a large sample of Chinese population in which men with SMI ≤ 40.8 cm^2^/m^2^ and women with SMI ≤ 34.9 cm^2^/m^2^ were considered to have low muscle mass [11]. The prevalence of low muscle mass in older adults with cancer was higher than in which without cancer according to previous researches [5]. In this study, 71 patients (28.7%) were considered with low muscle mass.

Whether low muscle mass is a risk factor of short-term postoperative complications in older patients is still controversial. Some studies showed that the surgical complications of patients with oesophageal cancer had no relation with low muscle mass [15], while in some other cancer such as lung cancer and renal cell carcinoma, low muscle mass seemed to be related with prognosis of surgery [16, 17]. This study suggested that low muscle mass was an independent risk factor of short-term postoperative complications in older patients with gastric cancer or colorectal cancer, and confirmed that low muscle mass is an important indicator of postoperative prognosis which suggests the necessity of preoperative diagnosis of low muscle mass. This research did not show any relationship between tumor stage, histologic type and surgical prognosis. A larger sample size may be needed for further clarification. In the multivariate logistic regression analysis, this research also showed that higher Charlson comorbidity index was risk factors for short-term surgical complications. A previous study showed that Charlson comorbidity index is an independent risk factor of short- and long-term mortality in hospitalized elderly patients and another one suggested that higher Charlson comorbidity index is related with postoperative complication and longer length of stay in patients with colorectal carcinoma [18]. This study was consistent with the previous research results. Low muscle mass was not found associated with the length of stay, while higher Charlson comorbidity index was a risk factor for longer length of stay, suggesting that patients with more underlying diseases should be taken better postoperative care.

Among the several factors related to low muscle mass found in this study, lower BMI and advanced age have been well discussed and recognized in previous studies [19]. But this research also found that higher BMI was a protective factor for low muscle mass, which may be somewhat controversial. Recently, many studies have come to a conclusion that obesity is a risk factor for low muscle mass as well, and obesity sarcopenia became a hot research topic [1]. However, there also have been many papers suggesting that obesity is not associated with low muscle mass [20], and obesity sarcopenia was not made a clear definition and diagnosis in the newly released sarcopenia guideline [1]. This study found that higher VFI is a risk factor for low muscle mass, while higher SFI is a protective factor for low muscle mass, that is, fat in different parts of the body has different effects on low muscle mass. Some research suggested that abdominal obesity is associated with the development of low muscle mass and parameter VSR is used as a parameter to define abdominal obesity [21]. VSR reflects differences in fat distribution, but cannot reflects the volume of fat. This study did not find any correlation between VSR and low muscle mass or postoperative outcomes. The value of VSR in low muscle mass needs further research efforts. The relationship between obesity and low muscle mass is still controversial and the mechanism is still unknown, which needs to be clarified by further studies. This study also found that the occurrence of low muscle mass was associated with a prior history of abdominal surgery which was not often mentioned in other studies. Abdominal surgery may lead to the functional decline of digestive system and results in emaciation.

This study had some limitations. The length of stay of patients with gastric cancer was found longer than those who with colorectal cancer, which may be because that gastric cancer and colorectal cancer have different surgical process and operative trauma. Cancer site was not found as a risk factor for low muscle mass which might be inconsistent as some clinical studies showed that patients with gastric cancer might have more weight loss than patients with colorectal cancer [22]. Since cancer cachexia is more severe in advanced stage and might not apparent in the early stage [23], and the ratio of patients in advanced stage in this research is not high, the result was not surprising and a larger sample size is needed. This study made analysis in patients with mixed gastrointestinal cancer but not separately, since the two kinds of tumor share some similarities in their characteristics. If with larger sample size, there should be further analysis separately in different cancer site and cancer stage. There was no follow-up of the long-term prognosis of the patients, such as long-term complications and quality of postoperative life. Due to the incompleteness of preoperative surgical examination, some possible relevant nutritional indicators, such as prealbumin, were not included in this study. Our team will continue investigating the subject.

## Conclusions

Age, lower BMI, and history of abdominal surgery are independent risk factors of low muscle mass in the older patients with gastrointestinal tumor. Low muscle mass and Charlson comorbidity index can predict the short-term prognosis of older patients undergoing gastrointestinal tumor resection. Precise preoperative evaluation is needed for the older patients with gastrointestinal tumor, and more attentive care should be taken to those with low muscle mass and higher Charlson comorbidity index to avoid short-term postoperative complications.

## Data Availability

The data in this study is not publicly available since national juridical restrictions, but further description or analysis of data are available from authors with reasonable request.

## Abbreviations

CT: Computed tomography
BMI: Body mass index
VFI: Visceral fat index
SFI: Subcutaneous fat index
WHO: World Health Organization
NLR: Neutrophils to lymphocytes ratio
UICC: Union for International Cancer Control
TNM: Tumor node metastasis
SMA: Skeletal muscle area
SFA: Subcutaneous fat areas
VFA: Visceral fat area
SMI: Skeletal muscle index
VSR: Visceral-to-subcutaneous ratio of fat area
OR: Odds ratio
CI: Confidence interval
AWGS: Asian Working Group for Sarcopenia
